# A mainstreaming oncogenomics model: improving the identification of Lynch syndrome

**DOI:** 10.3389/fonc.2023.1140135

**Published:** 2023-05-26

**Authors:** Rosie O’Shea, Ashley Crook, Chris Jacobs, Maira Kentwell, Margaret Gleeson, Katherine M. Tucker, Heather Hampel, Alanna Kulchak Rahm, Natalie Taylor, Sarah Lewis, Nicole M. Rankin

**Affiliations:** ^1^ Faculty of Medicine and Health, University of Sydney, Sydney, NSW, Australia; ^2^ Discipline of Genetic Counselling, Graduate School of Health, University of Technology Sydney, Sydney, NSW, Australia; ^3^ Parkville Familial Cancer Centre, Peter MacCallum Cancer Centre and Royal Melbourne Hospital, Parkville, VIC, Australia; ^4^ Department of Oncology, Royal Women’s Hospital, Parkville, VIC, Australia; ^5^ Hunter Genetics, Hunter Family Cancer Service, Newcastle, NSW, Australia; ^6^ Hereditary Cancer Clinic, Prince of Wales Hospital, Sydney, NSW, Australia; ^7^ Division of Clinical Cancer Genomics, Department of Medical Oncology and Therapeutics Research, City of Hope Comprehensive Cancer Center, Duarte, CA, United States; ^8^ Genomic Medicine Institute, Geisinger, Danville, PA, United States; ^9^ Cancer Research Division, Cancer Council NSW, Sydney, NSW, Australia; ^10^ Melbourne School of Population and Global Health, Melbourne University, Melbourne, VIC, Australia

**Keywords:** Lynch syndrome, routine genetic testing, oncology service delivery, mainstreaming, oncogenomics model

## Abstract

**Introduction:**

“Mainstreaming” is a proposed strategy to integrate genomic testing into oncology. The aim of this paper is to develop a mainstreaming oncogenomics model by identifying health system interventions and implementation strategies for mainstreaming Lynch syndrome genomic testing.

**Methods:**

A rigorous theoretical approach inclusive of conducting a systematic review and qualitative and quantitative studies was undertaken using the Consolidated Framework for Implementation Research. Theory-informed implementation data were mapped to the Genomic Medicine Integrative Research framework to generate potential strategies.

**Results:**

The systematic review identified a lack of theory-guided health system interventions and evaluation for Lynch syndrome and other mainstreaming programs. The qualitative study phase included 22 participants from 12 health organizations. The quantitative Lynch syndrome survey included 198 responses: 26% and 66% from genetic and oncology health professionals, respectively. Studies identified the relative advantage and clinical utility of mainstreaming to improve genetic test access and to streamline care, and adaptation of current processes was recognized for results delivery and follow-up. Barriers identified included funding, infrastructure and resources, and the need for process and role delineation. The interventions to overcome barriers were as follows: embedded mainstream genetic counselors, electronic medical record genetic test ordering, results tracking, and mainstreaming education resources. Implementation evidence was connected through the Genomic Medicine Integrative Research framework resulting in a mainstreaming oncogenomics model.

**Discussion:**

The proposed mainstreaming oncogenomics model acts as a complex intervention. It features an adaptable suite of implementation strategies to inform Lynch syndrome and other hereditary cancer service delivery. Implementation and evaluation of the model are required in future research.

## Introduction

Genomic testing (GT) strategies to identify those with hereditary cancer continue to expand. International evidence-based guidelines recommend that those with a significant cancer family history are offered germline GT and counseling (1–3). However, family history-based testing criteria result in suboptimal identification of hereditary cancer (4), and a high proportion of people who harbor pathogenic variants are not referred for GT even if they meet the testing criteria (4–10). Evidence for GT for those with particular tumor subtypes, e.g., epithelial ovarian cancer (11) and triple-negative breast cancer patients under 60 years (12), now allows direct access to GT in routine oncology care (mainstreaming), and guidelines are emerging for endometrial and colorectal cancers (13–15).

Genetic testing for colorectal cancer (CRC) targets deficient mismatch repair (dMMR) genes to identify Lynch syndrome (LS). In the CRC context, the dMMR genes *MLH1*, *MSH2*, *MSH6*, *PMS2*, or *EPCAM* are the first to be tested. However, limiting testing to these five genes can miss cases where heritability exists (16). Application of a wider 112-gene panel test with known and candidate genes for CRC in 274 patients found that 25% of cases had a pathogenic variant or variant of unknown significance that could potentially prove pathogenic in the future (17). Additionally, a 25-cancer gene panel testing used in a prospective study of early-onset CRC (<50 years) proved the benefit of this approach identifying 16% of patients (72 of 450) to have a pathogenic variant in at least one cancer predisposition gene (18). Similarly, a prospective cohort of 381 unselected endometrial cancer (EC) cases found that 9.2% had an LS gene and 3.4% had pathogenic variants in other cancer predisposition genes, with the application a 25-cancer gene panel (19). Multigene panel testing is emerging as a more effective approach but is challenged by the clinical utility and lack of evidence of disease causality in some genes included in the panel.

Identifying hereditary cancer and the significant higher risk of cancer development is important to enable access to early detection and risk-reducing measures at an earlier age, reducing cancer-related morbidity and mortality (19–23). Given the suboptimal identification of hereditary cancers, there is a lost opportunity for cancer prevention. Emerging opinion advocates for universal GT for hereditary breast and ovarian cancer (HBOC) and CRC and EC-associated LS at a general population level (24).

In the United Kingdom (UK), general population support exists for access to GT in determining the risk of ovarian cancer (25), CRC (26), and breast cancer at the population-level screening (27, 28). A positive attitude to a population-level GT exists when there are proven benefits, such as prevention and risk-reducing treatments (29).

In contrast, healthcare professionals are more cautious about population-based cancer GT. Fifty percent (166/330 for HBOC and 164/326 for LS responses) of the surveyed United States of America (USA)-based genetic counselors were reluctant to offer population-based GT (30). The main concern was health system readiness to implement large-scale GT. A further decade to prepare was highlighted in terms of workforce shortages, the need to upskill staff in genomics, and infrastructure barriers (31). However, public health initiatives to integrate genomics into health systems are emerging and build on initiatives to improve the identification of HBOC and LS initiated through the United States Preventive Services Task Force in 2005 and 2013 (32, 33).

In 2009, the Evaluation of Genomic Applications in Practice and Prevention recommended all CRC patients to undergo tumor screening for LS with follow-up GT if the screening tests were positive (34). A recent review using the Centers for Disease Control and Prevention Science Impact Framework (35) aimed to track the uptake of these recommendations through the five domains of the framework: disseminating science, creating awareness, catalyzing action, effecting change, and shaping the future (35). Practice change and widespread adoption of recommendations were initially slow to translate. However, after a decade of raising awareness through education, state cancer care planning, policy and national initiatives, and evolving evidence of genomic test utility, integration of screening and testing is beginning to emerge (35).

To accelerate genomics implementation into health services, the concept of a learning healthcare system came to life in 2007 (36) and was defined as an approach to allow for “science, informatics, incentives, and culture align for continuous improvement and innovation, with best practices seamlessly embedded in the delivery process and new knowledge captured as an integral by-product of the delivery experience” (36). Since 2015, the USA has taken the learning health care system approach to integrating genomics (37). The concept of complete learning in the system forges a continuum of clinical research aligned to care and quality improvement, allowing optimal care through continual adaptation to knowledge and evidence (37).

To assist in the translation of genomics into routine care, implementation frameworks to guide services have been utilized. The Implementation of Genomic Medicine Interventions in Clinical Care (IGNITE) framework (38) was developed and identified nine existing constructs from the Consolidated Framework for Implementation Research (CFIR) (39) deemed useful for genomics implementation. Seven additional constructs deemed important in genomics were developed to address patients, families, and communities (39). Further collaboration between IGNITE and the Clinical Sequencing Evidence-Generating Research consortium led to the development of the Genomic Medicine Integrative Research (GMIR) framework (40).

The GMIR framework consists of four domains that cover the organizational contextual factors, interventions, processes, and outcomes to investigate and build strategies for genomic clinical implementation depending on the disease context (40). The GMIR framework has been used in chronic kidney disease and rare undiagnosed pediatric disease contexts (40). However, to date, there is no specific conceptual oncogenomic clinical service model or strategy to guide the health system on the integration of genomic testing into routine oncology care.

With this gap in mind, we aimed to develop an implementation science-informed evidence-based conceptual mainstreaming oncogenomics clinical service model. With the future forecast for health system readiness for population-based cancer GT, our conceptual clinical service model provides generalizable implementation strategies scalable to the population-based level.

## Materials and methods

### Study design

**Figure 1:**
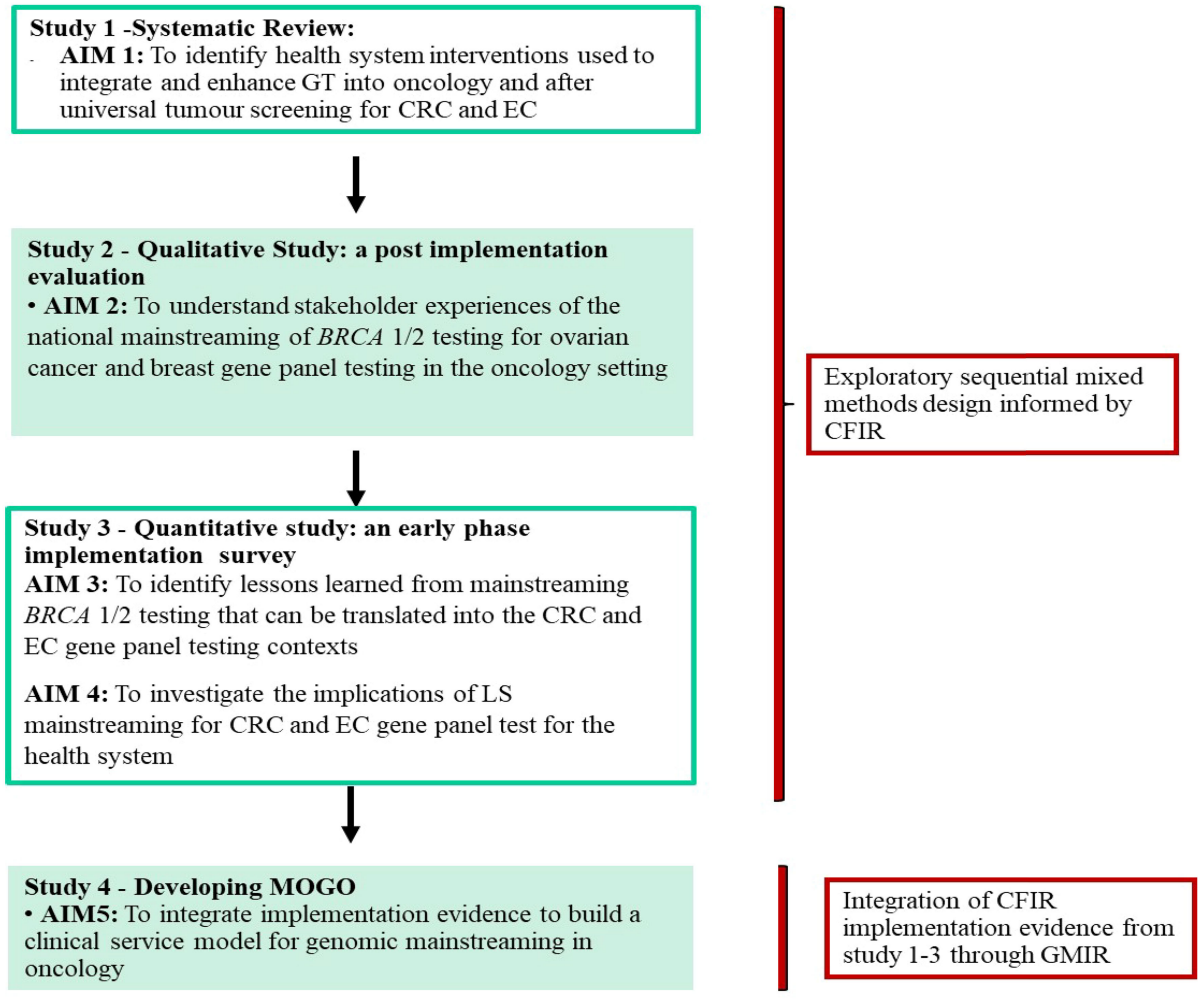
Study design flow for the Mainstreaming OncoGenomics mOdel (MOGO).

Three studies were completed to inform the development of the conceptual clinical service model (**Figure 1**). The model combines implementation evidence from a systematic review of GT programs in oncology care (41) (see **Supplementary Table 1** for the inclusion/exclusion criteria and the reference list of the included studies), a post-implementation *BRCA* mainstreaming qualitative study (42), and a quantitative survey with LS stakeholders about the potential implementation of GT mainstreaming in CRC and EC (43). We used an exploratory sequential mixed methods design (44). The systematic review was conducted to identify interventions that have been used to integrate GT into oncology services internationally with outcomes mapped to CFIR (39). The results from the CFIR-guided qualitative *BRCA* evaluation (42) were used in the design of an LS survey to focus on genomics implementation in the CRC and EC context (38). The full methods of each study are described in the primary publications (41–43).

### Data integration and model development

**Figure 2:**
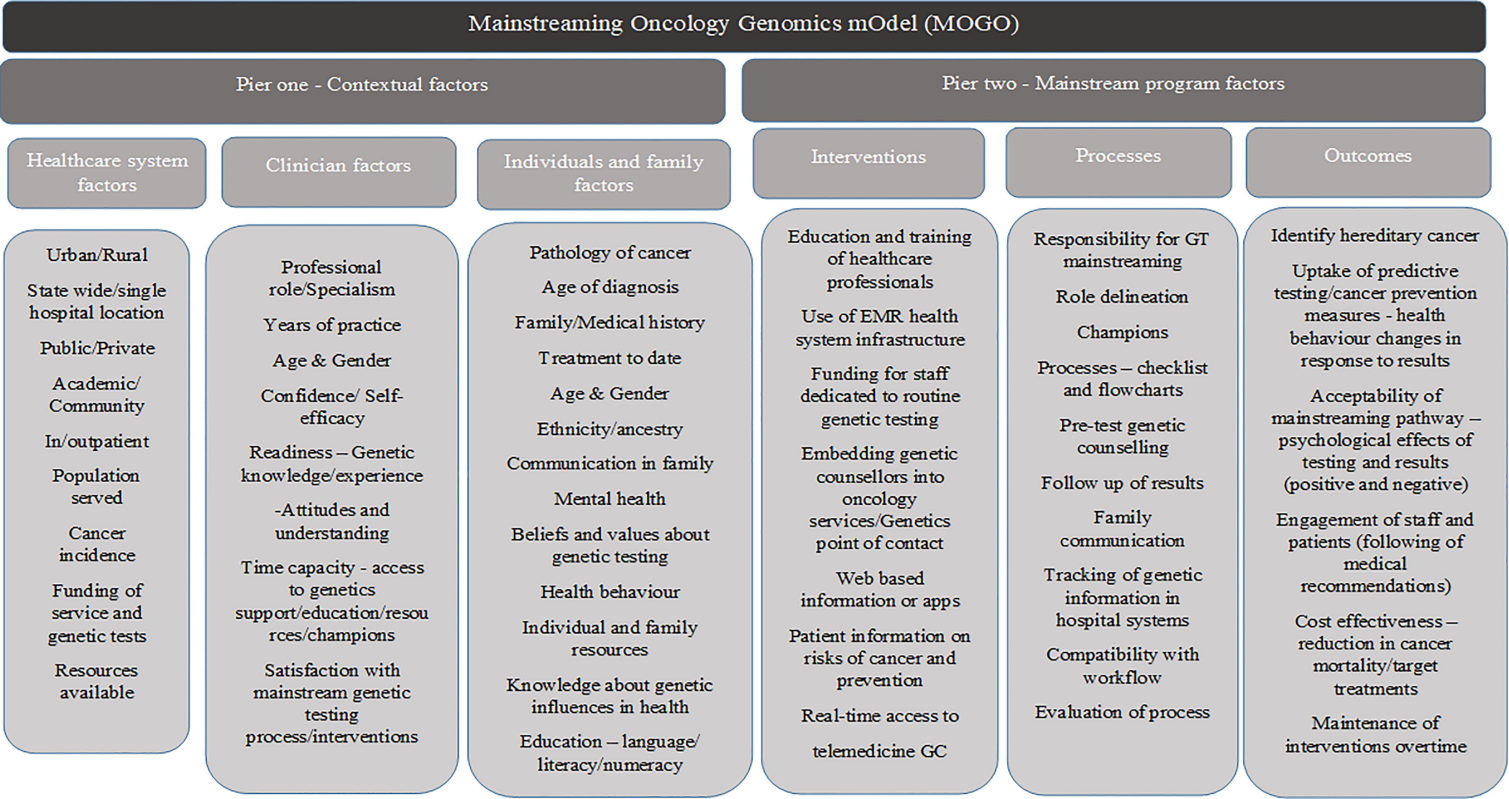
The GMIR-informed mainstreaming oncology genomics model for LS and other GT mainstreaming service delivery in oncology care.

The development of the conceptual clinical service model was through the building and generating of a model concept of mixed methods design and analysis (44). Data integration and analysis were through complementarity, where each set of methods was used to answer a series of questions for the purposes of evaluation and elaboration (45). The qualitative data from the *BRCA* mainstreaming evaluated key implementation factors that were then used to collect quantitative data to gain a broader understanding in the CRC and EC context. Complementarity of data was achieved by connecting qualitative, quantitative, and systematic review data through sequential dataset building informing the next study stage using the complementary CFIR domains and constructs. The qualitative (qual) and quantitative (quan) data were given the same level of importance and denoted (qual → quan) by the mixed methods notation system (46). Data transformation occurred at the analytical point of integration where the qual results were analyzed and, at a second stage, were then quantified. The connecting of qualitative to quantitative data allowed for narrative weaving by relating the qual and quan findings through a theme-by-CFIR construct basis, with the addition of synthesizing the systematic review findings. Finally, the details of the conceptual clinical service mainstreaming oncogenomics model were generated by matching the synthesized results from the narrative weaving to the four domains of the GMIR (40), either through contextual (health system, clinician, or individual/family factors) or mainstreaming program factors (interventions, process, or outcomes) (**Figure 2**).

## Results

### Systematic review

The systematic review (SR) identified 25 intervention studies used globally to integrate GT into oncology care (41) (Supplementary reference list). The CFIR complementary evidence used in the model development pertained to studies that measured patients’ or healthcare professionals’ satisfaction, belief, and feedback of the mainstreaming intervention, the engagement of health professionals through education, or implementing and executing the intervention according to an implementation plan. The intervention characteristics most used in the included studies to facilitate the integration of GT into oncology care were education followed by systems [i.e., electronic medical record (EMR) use and documentation of the process] and interdisciplinary practice (41). A classification of intervention components is described in more detail in the primary study (41). The specific SR results used in the model development are listed in **Table 1**.

Table 1Qualitative, quantitative, and systematic review data integration through the CFIR domain and construct and qualitative themes.

**Table:** 

Theme 1: Embracing the shift to mainstream genetic testing								
CFIR implementation influences of the inner setting and the implementation process	Subtheme	Qual→ (42)		Quan (43)				Systematic review (SR) (41)
		GHP *N* = 8 (%)	OHP *N* = 14 (%)	GHP *N* = 48 (%)	OHP *N* = 123 (%)	Pathologists *N* = 14 (%)		No. of intervention studies = 25
*Inner setting–implementation climate–relative priority*	*Clinical utility*	8/8 (100%)	10/14 (71%)	37/48 (77%)	92/117 (78%)	NA		8/25 (32%) Patients’ or healthcare professionals’ satisfaction with the mainstreaming intervention
*Implementation process*	*Current processes*	6/8 (75%)	11/14 (78%)	19/48 (39%)	84/123 (68%)	NA		24/25 (96%) Engaging health professionals through education/implementing the intervention 6/25 (24%) Executing implementation with a quality improvement/process model/implementation science framework
	*Future adaptation*	7/8 (87.5%)	7/14 (50%)	16/45 (35%)	67/115 (58%)	10/14 (71%)		3/25 (12%) Healthcare professionals’ feedback about the intervention

**Table:** 

Theme 2: Communication networks and role delineation needed to integrate genetic testing								
CFIR implementation influences of the inner setting	Subtheme	Qual→ (42)		Quan (43)				SR (41)
		GHP *N* = 8 (%)	OHP *N* = 14 (%)	GHP *N* = 48 (%)	OHP *N* = 123 (%)	Pathologists *N* = 14 (%)		No. of intervention studies = 25
*Inner setting–networks and communication and individuals involved*	*Communication networks*	8/8 (100%)	7/14 (50%)	27/45 (60%)	85/118 (72%)	13/14 (93%)		NA	
	*Role delineation*	7/8 (87.5%)	8/14 (57%)	36/42 (85%)	96/103 (93%)	12/12 (100%)		4/25 (16%) Healthcare professionals’ belief about their ability to undertake intervention
	*Genetics point of contact*	7/8 (87.5%)	8/14 (57%)	32/41 (78.0%)	74/102 (72.5%)	NA		NA	
	*Collaboration*	8/8 (100%)	8/14 (57%)	30/45 (66%)	98/112 (87.5%)	14/14 (100%)		NA

**Table:** 

Theme 3: Influencing factors on sustaining routine genetic testing								
CFIR implementation influences of the inner setting and the process	Subtheme	Qual→ (42)		Quan (43)				SR (41)
		GHP *N* = 8	OHP *N* = 14	GHP *N* = 48	OHP *N* = 123	Pathologist *N* = 14		No. of intervention studies = 25
*Inner setting–readiness for implementation–access to knowledge and information*	*Ongoing training needs*	8/8 (100%)	7/14 (50%)	42/44 (95%) 44/44 (100%)	100/103 (97%) 94/104 (90%)	NA		16/25 (64%) Use of education as a component of the intervention
*Inner setting–readiness for implementation–available resources*	*Resources and funding*	6/8 (75%)	7/14 (50%)	36/44 (81%)	76/113 (67%)	12/14 (85%)		24/25 (96%) Use of health professionals as a resource for implementation
*Process–engaging–champions*	*Genomics champions to sustain mainstreaming*	7/8 (87.5%)	6/14 (50%)	36/42 (85%)	89/104 (85%)	14/14 (100%)		NA	

NA, not applicable as data not present from the results of the original study.

GHP, genetic health professional; OHP, oncology health professional; SR, systematic review.

None of the included SR studies completed a comprehensive pre- and post-implementation assessment or evaluation, with less focus on implementation outcomes compared with client and service outcomes. These results informed the *BRCA* qualitative evaluative study design to understand the successes and challenges of *BRCA* mainstream implementation. The subsequent quantitative study evaluated the potential implementation factors for GT mainstreaming in the LS CRC and EC context.

### Qualitative and quantitative data integration

Of the 22 health professionals interviewed for the *BRCA* mainstreaming evaluation in subsets of breast and/or ovarian cancer (42) context, the majority embraced the shift to mainstream genetic testing due to clinical utility and streamlining the process for patient care (**Table 1**); for detailed quotes on this theme, refer to O’Shea et al. (42) Similarly, of the 158 completed and 27 partial LS survey responses from genetic health professionals (GHP) and oncology health professionals (OHP) in the CRC and EC context (43), the majority (77% of GHP and 78% of OHP) recognized the relative advantage of aligning GT with dMMR results and agreed that it would streamline care for CRC and EC patients (43) (**Table 1**).

Optimization of the *BRCA* mainstreaming process was recognized in the results delivery and follow-up phase. In both *BRCA* and CRC contexts, communication networks, role delineation, genetics point of contact, and collaboration were key components of the organization’s inner setting that need to function well for mainstreaming to be adopted and maintained. Ongoing training, resources and funding, and champions were highlighted as key measures to sustain mainstreaming into the future (42, 43).

In both contexts, overcoming barriers was recognized, with genomics champions regarded as essential to integrate a genetic testing pathway. Facilitators such as embedding genetic counselors into an oncology clinic, multidisciplinary team documentation, and tracking to follow-up test results along with EMR tracking and flowcharts or checklists for an understandable mainstreaming pathway or process were also identified (**Table 2**). Many other suggested interventions such as online training, automatic or e-mail reminders, information provision, applications, and telehealth were viewed as suitable to facilitate the adoption of mainstreaming into routine care (**Table 2**).

**Table 2:** Suggested interventions to overcome barriers to mainstream genetic testing in oncology services.

CFIR construct—intervention characteristics (personnel or education)					CFIR construct—intervention characteristics (systems and engaging)				
Suggested intervention	No./total (%)				Suggested intervention	No./total (%)			
	GHP		OHP			GHP		OHP		
	Qual→ (42)	Quan (43)	Qual→ (42)	Quan (43)		Qual→ (42)	Quan (43)	Qual→ (42)	Quan (43)
A genetics healthcare professional available *via* telephone for ongoing support when integrating panel genetic testing into routine practice	7/8 (87%)	32/41 (78%)	8/14 (57%)	74/102 (72%)	Multidisciplinary team meeting template to include genetic tests ordered and the need for follow-up discussed at the meeting	1/8 (12%)	34/41 (82%)	6/14 (42%)	68/102 (66%)
Embedded genetic counselor in oncology to do pre-test genetic counseling	7/8 (87%)	29/41 (70%)	7/14 (50%)	69/102 (67%)	Checklist or flowchart of the process for integrating genetic panel testing as standard practice	2/8 (25%)	30/41 (73%)	4/14 (28%)	61/102 (59%)
Information for oncology health professionals (OHP) on how to talk with their patients about genetics and genetic testing	1/8 (12%)	27/41 (65%)	2/14 (14%)	60/102 (58%)	Patient tracking system in the EMR to ensure genetic results are followed up	3/8 (37%)	28/41 (68%)	6/14 (42%)	63/102 (61%)
Online training regarding panel genetic testing and adoption as standard practice	2/8 (25%)	26/41 (63%)	1/14 (7%)	56/102 (54%)	An easy way to order genetic tests and log the test order in the electronic medical record (EMR)	NA	24/41 (58%)		81/102 (79%)
Face-to-face education on genetics and panel genetic test adoption	NA	25/41 (60%)	NA	38/102 (37%)					
Genetic-specific training in medical school or oncology training	NA	33/41 (80%)	NA	53/102 (51%)					
Information for OHP about patient management if a test is positive	NA	25/41 (61%)	NA	70/102 (68%)	Integration of genetic information into the main EMR system	NA	16/41 (39%)	NA	48/102 (47%)
Information for OHP about how to manage questions from family members about their genetic risk	NA	21/41 (51%)	NA	63/102 (61%)	An app with all the relevant information to integrate genetic testing into my practice	NA	11/41 (26%)	2/14 (14%)	47/102 (46%)
					An app with patient friendly information about genetic testing	NA	15/41(36%)	NA	50/102(49%)
Handouts for OHP to give their patients specific information if a test is positive	NA	21/41 (51%)	NA	83/102 (81%)	A website with all the relevant information to integrate genetic testing into my practice	NA	18/41 (43%)	NA	63/102 (61%)

NA, not applicable as data not present from the results of the original study.

In summary, both the qual and quan datasets confirmed the importance of role delineation, organization readiness, genetics support, education, and identification of interventions to overcome barriers for mainstreaming sustainability in oncology services. Adaptation of the *BRCA* mainstreaming intervention was recognized to ensure a practical fit with the process and to support LS stakeholder consultation in the context of CRC and EC to plan and evaluate the process. Solutions were identified to overcome barriers along with the education, system, documentation, and interdisciplinary practice solutions trialed globally to mainstream GT as identified by the systematic review (41). A specific focus on the implementation and evaluative outcomes of GT mainstreaming programs was lacking and is critical to measure in future programs. The above evidence was combined and matched to the four domains of the GMIR (40) to inform the mainstreaming oncogenomics conceptual clinical service model to guide the translation of GT into oncology services.

### Model development

#### General content

We created a conceptual clinical service model that integrates the GMIR (40) contextual implementation factors, interventions, processes, and outcomes that were found to be important to GT mainstreaming in the oncology setting from our research. The Mainstreaming OncoGenomics Model (MOGO) consists of two piers with interlinking components (**Figure 2**). The first pier comprises three components describing the organizational factors important to characterize the structure and function of the health system and the clinicians and patients engaged with the system and services. The components of the contextual factors in pier 1 influence GT mainstreaming program design factors in pier 2 through processes, interventions, and outcome measures in context-specific health system organizations. Our intention is that the conceptual clinical service model can guide health organization planning and developing LS and other GT mainstreaming programs in oncology. It does not intend to be prescriptive and highlights the important contextual factors to characterize and suggest interventions, processes, and outcomes to facilitate mainstreaming programs across cancer types. It can act as a single complex intervention unit or an adaptable conceptual model with a suite of implementation strategies to be described and tested in a health system.

#### Pier 1—contextual factors

The first component of this pier begins with defining the structure and function of the health system or organization where the oncology GT mainstreaming program is being introduced. The health system can be characterized in terms of the type of system or organization, the population served, and cancer incidence. The general description would include reporting on the setting, location, service structure, resources and funding, and the population served. The description of the system is the basis for the study of the related components, i.e., how the clinicians interact in this environment and the resources available to them and how the population being served engage with the system and services offered.

The second component of the health professional factors examines the professional role of those undertaking the GT mainstreaming initiative, along with their years of practice, age, and gender. These factors can impact clinician confidence and self-efficacy to take on mainstreaming, which is linked to their readiness. Clinician readiness extends to their knowledge and experience of genetics, along with their attitudes and understanding of its use. The time capacity to take on a GT mainstreaming role along with access to support and how satisfied they are with the new mainstreaming process in their workflow are important factors to consider.

The third component of this pier is the patient population being served by the organization, characterized by the type of cancer, age, treatment, and medical and family history among others (see **Figure 2**). GT will be integrated into multiple cancer types, and the context of cancer diagnosis at pathology, communication to the medical team, and workflow to determine GT eligibility require adaptation and description when the model is applied in health systems. Familial factors to consider would be family communication, depression, anxiety, or stress (mental health) with family issues or cancer diagnosis. Patient factors that could impact GT uptake such as belief, value, or knowledge of GT and its influence on health could interact with the health behavior impact. It is important to understand the system, clinician, and patient factors that can impact the mainstreaming program design, potential implementation success, and individual outcomes.

#### Pier 2—mainstreaming program factors

Three aspects of this pier encompass how the oncology GT mainstream program can be delivered addressing the health system and clinician needs. The first and second components describe interventions and processes to consider for individuals or systems and process planning that can allow routine pre-test genetic counseling and test ordering to be integrated into oncology. Some of the main elements to consider in a mainstreaming program are as follows: the set of interventions to support GT integration and decisions about how to obtain informed consent through pre-test genetic counseling in oncology, for a specific cancer type based on eligible criteria and guidelines; and the process of integrating genetic healthcare in oncology, for example, through an oncology-led patient pathway, i.e., the process from the patients’ initial contact with oncology services to their OHP giving access to GT either confirming or excluding a diagnosis of hereditary cancer and informing treatment management. An iterative learning healthcare systems approach to adapt to the mainstreaming processes by routinely evaluating the MOGO outcomes must be taken into consideration. These can be measured through cancer prevention, reduction in mortality, and cost utility with targeted treatments, along with the acceptability and amount of engagement from staff and patients. Adjusted processes can allow for optimal outcomes and sustained adoption. The specific aspects to consider are described in **Figure 2**.

Pier 2-related components intersect with pier 1 contextual factors from a healthcare system, clinician, individual, and family perspective. Analysis of contextual factors allows preparation for the mainstreaming program factors such as the expected demand for such a program through the population served, the number and type of cancers diagnosed, and the resources needed to implement such programs. Barriers are important contextual pier 1 factors to be identified and can be overcome in pier 2 by designing suitable interventions as indicated in **Figure 2**. With a learning healthcare systems approach, pier 2 (component 3) highlights outcome measures to be evaluated to ensure understanding of the need for adaptation of the mainstreaming oncology genomics programs to ensure sustainability.

## Discussion

The aim of this paper was to develop a conceptual mainstreaming oncogenomics clinical service model to inform current and future oncology GT mainstreaming programs. Our genomics implementation evidence from a systematic review and qualitative and quantitative data focusing on genomic test integration into oncology services informed the content of the MOGO. Using GMIR as a guiding framework for genomics implementation research, the MOGO can be used as a generalizable foundation for the design and development of LS and other GT mainstreaming programs in oncology and should serve as a scalable learning canvas for future population-based cancer GT. The information contained in the clinical service model highlights the importance of the health system context and readiness and the interventions, process infrastructure, and funding needed to integrate GT mainstreaming programs into the future.

The MOGO consists of two piers to consider in the design and development of LS and other GT mainstream programs. The success and sustainability of a mainstreaming program first lie in understanding the organizational contextual factors (pier 1). The contextual recognition of genomic-specific factors for service delivery was highlighted by the IGNITE model, which extended CFIR to recognize the unique features that genomics information have on the family system (38). The IGNITE patient domain focused on extending the model to recognize the contribution of patient views, emotions, and attitudes toward genetic testing especially in the context of unaffected family members and their community network. The MOGO factors in these facets in the “individual and family factors” domain were informed by our systematic review finding of a lack of patient evaluative outcomes, with only 24% of studies focusing on the patient’s satisfaction of the mainstreaming intervention (41). The MOGO includes evaluative components to address genomics implementation through patient’s experience, satisfaction, health behavior, and overall engagement with the program.

The MOGO recognizes how clinicians’ factors such as self-efficacy, knowledge, and confidence (pier 2) intertwine with the educational interventions in pier 2 to ready health professionals with the required information to take on the routine pre-test genetic counseling role in oncology. Studies with OHP found that self-efficacy, knowledge, and confidence are the recognized barriers to the integration of GT in mainstream medicine (47). Emerging literature shows cancer specialists’ positive attitudes toward the use of genetic information in precision medicine, with 63.7% favoring its use in treatment and prognostic information but with concerns over cost, knowledge, potential misuse, access, and results delivery (48). To overcome clinician factor barriers, solutions such as a designated point of contact from a genetic service to alleviate concerns, education, or embedding a genetic counselor into routine oncology care are recognized in MOGO. The benefits of these mainstreaming approaches are evident in the USA and Australia (49–51), revealing more patients getting timely access to GT and appropriate follow-up in the system.

The evaluative outcomes highlighted in the MOGO (pier 2) ensure that a learning healthcare system concept of genomics implementation can be taken. Initial intervention design and process planning require real-world system trial and adaptation to achieve optimal outcomes for the patient and the system. A ProvenCare initiative in cancer care by the Geisinger Health System used evidence-based care guidelines to devise protocols and electronic health record (EHR) implementation strategies (52). Success was achieved through a 90% uptake of patient care elements in six hospital sites (52). Dashboard analytics to assess the uptake of the protocol in practice, followed by tracking of outcomes for lung cancer patients, led to the provision of optimal care and translation of the latest evidence into practice (52). Monitoring of future mainstreaming initiatives with EHR infrastructure could allow for similar learning and adaptation to enhance success.

The MOGO recommends the use of the EHR as an important tool in intervention design and reporting of outcomes in GT mainstreaming programs. Several studies in our systematic review used the EHR for streamlining appointments, checking all patients have access to GT, notifying clinicians involved in the genomic healthcare of their patients, and tracking of results to ensure appropriate follow-up (41). An organization’s EHR infrastructure along with clinician, laboratory, and IT collaboration led to the successful EHR integration of genetic information in one US institution (53). This integration allowed for ordering of genetic tests directly in the EHR with results sent promptly into the system and used to notify the clinician.

The above EHR initiatives are examples of how the interventions identified in the MOGO could be operationalized in the oncology context and feed into an evaluative loop assessing patient outcomes such as hereditary cancer identification, cancer prevention through uptake of screening or risk-reducing measures and predictive testing in families, and the utilization of targeted treatments. These outcomes can then be used in a robust cost-effectiveness evaluation of GT mainstreaming programs.

The strength of the MOGO is its underpinning of broad evidence from various health professionals, hospital organizations, disease contexts, and GT mainstreaming intervention evaluations to inform the various components. MOGO was informed by global studies (UK, USA, Australia, and Europe) from the systematic review of evidence encompassing intervention characteristics and evaluation from diverse system structures and contexts. The sequential mixed methodology of building datasets designed with CFIR and model development with data integration and analysis using complimentary CFIR domains and constructs allowed for robust implementation science data to be generated across cancer types and systems. For this reason, the evidence-informed model allows for generalizability across systems and cancer types. The model recognizes the importance of planning, consultation, and role delineation needed for different cancer contexts considering the hospital system infrastructure and resources available. The MOGO intends to be a resource for the system and health professionals designing LS and other GT mainstreaming programs and can be adapted to the inner setting of organizations or individual characteristics to ensure flexibility. The adoption of a MOGO for the evaluation of different GT mainstreaming programs would allow comparative lessons to be learned in diverse programs and in comparison with other countries.

The limitations of the MOGO exist in the lack of direct evidence to inform a social determinant component for contextual factors informing policy, systems, and education needs of an organization and the population it serves. The mixed methodology allows for depth of implementation evidence but the time required to gather and build this evidence to inform future mainstreaming program design may be prohibitive in resource-limited organizations and to keep pace with a rapidly evolving field. The qualitative and quantitative evidence may not represent all stakeholders involved in mainstreaming contexts for breast, ovarian, CRC, and EC, and future implementation research design using hybrid effectiveness methods could facilitate iterative service designs with all stakeholders.

Future research into GT mainstreaming programs needs to focus on the populations’ access and uptake of genetics within oncology services and evaluate the communities’ knowledge about genetic influences in their healthcare. Social policy integrating information about GT into general public education and campaigns could facilitate broader public knowledge, focusing on the laws and regulations regarding the use of genetic test information in individuals and in family healthcare decisions. As a population-based cancer GT emerges, future research in social policy and public needs is important. The qualitative and quantitative evidence may not represent all stakeholders involved in mainstreaming contexts for breast, ovarian, colorectal, and endometrial cancer outside of Australia. Therefore, the MOGO needs to be assessed and trialed by oncology and genetic experts in the field. The outcome domains of the MOGO require future research to identify or create validated measures to evaluate genomic outcomes consistently in oncology.

## Data availability statement

The original contributions presented in the study are included in the article/**Supplementary Material**. Further inquiries can be directed to the corresponding author.

## Ethics statement

The studies involving human participants were reviewed and approved by the University of Sydney. The patients/participants provided their written informed consent to participate in this study.

## Author contributions

RO’S, NR, and SL contributed to the conception and design of the study. RO’S performed the analysis. RO’S wrote the first draft of the manuscript. All authors contributed to the article and approved the submitted version.
