# Supplementary material for: A mainstreaming oncogenomics model: improving the identification of Lynch syndrome
Source: Front Oncol. 2023 May 26;13:1140135. doi: 10.3389/fonc.2023.1140135 (PMC10256118; doi:10.3389/fonc.2023.1140135)
Supplement: Supplementary file 1 [file Table_1.docx]

**Supplementary material – Table 1 and references taken from original primary study (O’Shea et al 2021^41^)**

**Table 1. Inclusion and exclusion criteria for routine genetic testing integration intervention studies in oncology**

| **Selection criteria** | **Inclusion criteria** | **Exclusion criteria** |
| --- | --- | --- |
| **Study type** | Interventions |  |
| **Study design** | Randomised control trials (RCTs) – including step wedge and cluster RCT  Non-randomised quasi-experimental design  -Cohort study  -Before and after study (including  interrupted time series and multiple baseline design)  Observational studies  -cohort studies  -Case series for intervention outcomes only  Qualitative-  Qualitative studies that report on implementation outcomes | Case reports, case series, case-control, cross-sectional, designed with no comparator  Cross-sectional: Single point in time knowledge study no before or after (no comparison)  Case series (no comparator) except if report on an intervention outcome  Qualitative studies if they do not report on implementation or intervention outcomes |
| **Population** | Health providers of genetic testing and/or counselling for HBOC/LS including (but not limited to);   - genetic counsellors, - clinical geneticists, - oncologists   FOR  Adult patients (>18 years old) diagnosed with the following cancers;   - ovarian - breast - colorectal - endometrial   Minimum of 80% of population has to have the above cancers | Health providers with no involvement in mainstreaming genetic testing  Patients with other cancers not related to hereditary cancer syndromes HBOC and hereditary colorectal cancer LS  No specific data for the subgroup of interest  Paediatric cancer patients <18 years  Asymptomatic individuals at high risk of HBOC and LS  Asymptomatic relatives of HBOC and LS identified families |
| **Intervention** | Interventions aiming to implement pre-test genetic counselling and genetic or genomic testing through mainstreaming* for breast and ovarian cancer  OR  Interventions to increase pre-test genetic counselling and genetic testing completion rates after universal tumour screening (UTS) for colorectal and endometrial cancer.  For example, through increasing;  - the knowledge/awareness of health providers re HBOC or LS  - patient access to genetic testing  - identification of hereditary cancer such as HBOC and LS  - follow up of HBOC/LS patients getting through the health system  OR  Multicomponent interventions that target the health provider and patient to achieve the above | Interventions not used to increase identification of HBOC or LS  Research genetic or genomic testing  Laboratory methods of genetic testing  Data on likelihood of HBOC/LS mutation detection, mutation incidence or phenotype without any information on mainstreaming of genetic testing for the patient or health provider  Childhood-onset hereditary cancer  Multi component interventions aimed  solely at the patient except if the patient intervention is targeted to influence the health system  Studies with UTS steps not involved in mainstreaming  Physician discretionary referral to genetic counselling |
| **Comparator** | Standard care/no intervention  Another Intervention |  |
| **Outcomes** | *Implementation* *Outcomes*–  -Acceptability  -Adoption  -Appropriateness  -Feasibility  -Cost  -Fidelity  -Penetration  -Sustainability  *Service Outcomes*  -Efficiency  -Safety  -Effectiveness  -Equity  -Patient centeredness  -Timeliness  *Client outcomes*  -Satisfaction  -Function  -Symptomatology  **CFIR**  -Intervention Characteristics  -Inner Setting  -Outer Setting  -Characteristics of Individuals  -Process | Outcomes not linked to mainstreaming of genetic or genomic testing or enhancing the uptake of universal tumour screening to improve identification of HBOC and LS |
| **Language** | English | Not in English |
| **Publication period** | From January 1^st^ 1980 - present | Before 1980 |
| **Publication type** | Journal article | Conference proceedings, posters, comments or editorials, letters, news, editorials, narrative reviews, theses, review |

- For the purposes of this systematic review mainstreaming is the process where all patients with a particular cancer are offered direct access to genetic testing in oncology care through pre-test genetic counselling regardless of who does the genetic counselling (eg. could be specialist or genetic counsellor).

**Supplementary References: Included systematic review studies**

29. George A, Riddell D, Seal S, Talukdar S, Mahamdallie S, Ruark E, Cloke V, Slade I, Kemp Z, Gore M, Strydom A, Banerjee S, Hanson H, Rahman N. Implementing rapid, robust, costeffective, patient-centred, routine genetic testing in ovarian cancer patients. Sci. Rep. 2016; doi: 10.1038/srep29506

30. Percival N, George A, Gyertson J, Hamill M, Fernandes A, Davies E, Rahman N, Banerjee S. The integration of BRCA testing into oncology clinics British Journal of Nursing. 2016;12:690-694

31. Plaskocinska I, Shipman H, Drummond J, Thompson E, Buchanan V, Newcombe B, Tischkowitz, M . New paradigms for BRCA1/BRCA2 testing in women with ovarian cancer: results of the Genetic Testing in Epithelial Ovarian Cancer (GTEOC) study. J Med Genet. 2016; doi:10.1136/jmedgenet-2016-10390

32. Rahman B, Lanceley A, Kristeleit RS, Ledermann, JA, McCormack M, Mould T, Side L. Mainstreamed genetic testing for women with ovarian cancer: first-year experience. J Med Genet 2019; 56: 195–198.

33. Kentwell M, Dow E, Antill Y, Wrede C D, McNally O, Higgs E, Hamilton A, Ananda S, Lindeman GJ, Scott CL. Mainstreaming cancer genetics: A model integrating germline BRCA testing into routine ovarian cancer clinics. Gynecologic Oncology. 2017 145: 130-136

34. Senter L, O'Malley DM, Backes FJ, Copeland LJ, Fowler JM, Salani, R, Cohn DE. Genetic consultation embedded in a gynecologic oncology clinic improves compliance with guideline-based care. Gynecologic Oncology. 2017; 147: 110–114

35. Bednar EM, Oakley HD, Sun CC, Burke CC, Munsell MF, Westin SN, Lu KH. A universal genetic testing initiative for patients with high-grade, non-mucinous epithelial ovarian cancer and the implications for cancer treatment. Gynecologic Oncology. 2017;146 399–404

36. Uyar D, Neary J, Monroe A, Nugent M, Simpson P, Geurts JL. Implementing a quality improvement project for universal genetic testing in women with ovarian cancer. Gynecologic oncology. 2018 doi 10.1016/j.ygyno.2018.03.0590090-8258/

37. Swanson CL, Kumar A, Maharaj JM, Kemppainen JL, Thomas BC, Weinhold MR, Slaby KM, Mara KC, Wick MJ, Bakkum-Gamez, JN. Increasing genetic counseling referral rates through bundled interventions after ovarian cancer diagnosis. Gynecologic Oncology. 2018; 149: 121–126

38. Brown J, Athens A, Tait DL, Crane EK, Higgins RVR, Naumann W, Hadzikadic Gusic L, Amacker-North, L. A Comprehensive Program Enabling Effective Delivery of Regional Genetic Counseling. Int J Gynecol Cancer 2018;28: 996-1002

39. Bednar EM, Sun CC, Camacho B, Terrell J, Rieber AG, Ramondetta L, Freedman RS, Lu KH. Disseminating universal genetic testing to a diverse, indigent patient population at a county hospital gynecologic oncology clinic. Gynecologic Oncology. 2019;152: 328–333

40. Kemp Z, Turnbull A, Yost S, Seal S, Mahamdallie S, Poyastro-Pearson E, Warren-Perry M, Eccleston A, Tan M, Hwang Teo S, Turner N, Strydom A, George A, Rahman N. Evaluation of Cancer-Based Criteria for Use in Mainstream BRCA1 and BRCA2 Genetic Testing in Patients With Breast Cancer. JAMA Network Open. 2019; e194428. doi:10.1001

41. Miesfeldt S, Feero WG, Lucas FL, Rasmussen K. Association of patient navigation with care coordination in a Lynch syndrome screening program. Transl Behav Med. 2018 23;8:450-455.

42. Long JC, Debono D, Williams R, Salisbury E, O’Neill S, Eykman E, Butler J, Rawson R, Phan-Thien K, Stephen R. Thompson SR, Braithwaite I, Chin JM, Taylor N. Using behaviour change and implementation science to address low referral rates in oncology. BMC Health Services Research. 2018 18:904

43. Cohen PA, Nichols CB, Schofield L, Van Der Werf S, Pachter N. Impact of Clinical Genetics Attendance at a Gynecologic Oncology Tumor Board on Referrals for Genetic Counseling and BRCA Mutation Testing. International Journal of Gynecological Cancer. 2016; 26: 892-897

44. Heald B Plesec T, Liu X, Pai R, Patil D, Moline J, Sharp RR, Burke CA, Kalady MF, Church J, Eng C. Implementation of Universal Microsatellite Instability and Immunohistochemistry Screening for Diagnosing Lynch Syndrome in a Large Academic Medical Center. Journal of Clinical Oncology. 2013; 31: 1336-1340

45. Hanley GE, McAlpine JN, Miller D, Huntsman D, Schrader KA, Gilks CB, Mitchell G. A population-based analysis of germline BRCA1 and BRCA2 testing among ovarian cancer patients in an era of histotype-specific approaches to ovarian cancer prevention. BMC Cancer. 2018; 18:254 <https://doi.org/10.1186/s12885-018-4153-8>

46. Petzel SV, Vogel RI, McNiel J, Leininger, A, Argenta, PA, Geller MA. Improving Referral for Genetic Risk Assessment in Ovarian Cancer Using an Electronic Medical Record System. International Journal of Gynecological Cancer. 2014; 24:1003-1009

47. Cohen SA, Laurino M, Bowen DJ, Upton MP, Pritchard C, Grady WM. Initiation of Universal Tumor Screening for Lynch Syndrome in Colorectal Cancer Patients as a Model for the Implementation of Genetic Information Into Clinical Oncology Practice. Cancer. 2016; 393-401

48. Tutty E, Petelin L, McKinley J, Young M, Meiser B, Rasmussen VM, Forbes Shepherd R, James PA, Forrest LE. Evaluation of telephone genetic counselling to facilitate germline BRCA1/2 testing in women with high-grade serous ovarian cancer. European Journal of Human Genetics. 2019; doi.org/10.1038/s41431-019-0390-9

49. Meiser B, Gleeson M, Kasparian N, Barlow-Stewart K, Ryan M, Watts K, Menon D, Mitchell G, Tucker K. There is no decision to make: Experiences and attitudes toward treatment-focused genetic testing among women diagnosed with ovarian cancer. Gynaecologic Oncology. 2012; 124: 153-157

50. Shipman H, Flynn S, MacDonald-Smith CF, Brenton J, Crawford R, Tischkowitz M, Hulbert-Williams NJ. Universal BRCA1/BRCA2 Testing for Ovarian Cancer Patients is Welcomed, but with Care: How Women and Staff Contextualize Experiences of Expanded Access. J Genet Counsel. 2017; 26:1280–1291

51. McLeavy L, Rahman B, Kristeleit R, Ledermann J, Lockley M, McCormack M, Mould T, Side L, Lanceley A. Mainstreamed genetic testing in ovarian cancer: patient experience of the testing process. Int J Gynecol Cancer 2020;30:221–226.

52. Rumford M, Lythgoe M, McNeish I, Gabra H, Tookman L, Rahman N, George A, Krell J. Oncologist-led BRCA ‘mainstreaming’ in the ovarian cancer clinic: A study of 255 patients and its impact on their management. Sci Rep Nat Res 2020 10:3390

53. Grindedal EM, Jørgensen K, Olsson P, Gravdehaug B, Lurås H, Schlichting E, Vamre T, Wangensteen T, Heramb C, Mæhle L. Mainstreamed genetic testing of breast cancer patients in two hospitals in South Eastern Norway. Familial Cancer 2020; 19:133–142

54. Richardson M, Jung Min H, Hong Q, Compton K, Wing Mung S, Lohn Z, Nuk J, McCullum M, Portigal-Todd C, Karsan A, Regier D, Brotto LA, Sun S, Kasmintan A, Schrader KA. Oncology Clinic-Based Hereditary Cancer Genetic Testing in a Population-Based Health Care System. Cancers 2020;12:338

55. Lobo M, López-Tarruella S, Luque S, Lizarraga S, Flores-Sánchez C, Bueno O, Solera J, Jerez Y, González del Val R, Palomero MI, Cebollero M, Echavarría I, Torres G, Martín M, Márquez-Rod I. Evaluation of Breast Cancer Patients with Genetic Risk in a University Hospital: Before and After the Implementation of a Heredofamilial Cancer Unit. Journal of Genetic Counseling 2018; 27:854–862
